# New Multitarget Approaches in the War Against Glioblastoma: A Mini-Perspective

**DOI:** 10.3389/fphar.2018.00874

**Published:** 2018-08-03

**Authors:** Simona Sestito, Massimiliano Runfola, Marco Tonelli, Grazia Chiellini, Simona Rapposelli

**Affiliations:** ^1^Department of Pharmacy, University of Pisa, Pisa, Italy; ^2^Biochemistry Department, University of Wisconsin-Madison, Madison, WI, United States; ^3^Department of Pathology, University of Pisa, Pisa, Italy; ^4^Interdepartmental Research Centre for Biology and Pathology of Aging, University of Pisa, Pisa, Italy

**Keywords:** glioblastoma, multitarget drug, multiple kinase inhibitor, small molecules, combination therapy

## Abstract

Glioblastoma multiforme (GBM) is the most common tumor of the CNS, and the deadliest form of brain cancer. The rapid progression, the anatomic location in the brain and a deficient knowledge of the pathophysiology, often limit the effectiveness of therapeutic interventions. Current pillars of GBM therapies include surgical resection, radiotherapy and chemotherapy, but the low survival rate and the short life expectation following these treatments strongly underline the urgency to identify innovative and more effective therapeutic tools. Frequently, patients subjected to a mono-target therapy, such as Temozolomide (TMZ), develop drug resistance and undergo relapse, indicating that targeting a single cellular node is not sufficient for eradication of this disease. In this context, a multi-targeted therapeutic approach aimed at using compounds, alone or in combination, capable of inhibiting more than one specific molecular target, offers a promising alternative. Such strategies have already been well integrated into drug discovery campaigns, including in the field of anticancer drugs. In this miniperspective, we will discuss the recent progress in the treatment of GBM focusing on innovative and effective preclinical strategies, which are based on a multi-targeted approach.

## Introduction

Despite the recent advantages in the field of drug design, the development of innovative and selective single-target drugs seems to be a task of little success, at least in the field of anticancer drug research. Too often, therapies properly designed to act selectively against a single-target display a low or no efficacy. Indeed, the cellular processes cannot be effectively modulated by a single-target drug and this weakness can be attributed to the complexity of pathways and molecular alterations implicated in the development and progression of cancer. The most tangible evidence of the failure of one-drug-one-target therapy is observed in tumor forms that are particularly refractory and resistant to chemotherapies, such as glioblastoma multiforme (GBM). GBM is the most common tumor of the CNS and it is considered as the deadliest form of brain cancer with a survival rate of less than 4% (Ohgaki and Kleihues, 2013). Moreover, the average survival rate in GBM patients undergoing maximum safe surgical resection is approximately only 14 months. When surgery is followed by adjuvant postoperative multimodal therapy, including both chemo- and radiotherapy with conventional cytotoxic agents (Delgado-López and Corrales-García, 2016), the resulting life expectation increases to about 5 years. Such a poor prognosis for GBM has been related to various factors, such as the rapid onset of the disease, cancer location, and a deficient knowledge of the pathophysiology (Carlsson et al., 2014), that limit the availability of efficient therapeutic tools, thus highlighting the urgency to find novel efficacious treatments.

Among the great number of key signaling pathways involved in GBM, the PI3K/Akt/mTOR pathway is one of the most investigated and targeted, since it regulates several cellular processes, including protein synthesis, proliferation, apoptosis, angiogenesis, and migration (Li et al., 2016). EGFR, the upstream activator of this pathway, is considered a common driver of GBM progression, as it is mutated in 40% of all GBM cases (Hatanpaa et al., 2010). EGFR substantially promotes the activation of the downstream PI3-Kinase. This activation, however, also occurs independently of EGFR, either through gain-of-function mutations in PIK3CA or by a PTEN deregulation (McLendon et al., 2008), which plays a pivotal role as signal suppressor in the PI3K/Akt/PDK1 pathway. Similarly, protein p53, the guardian of genome, is strongly associated with GBM. The p53 gene mutation is linked to the transition from low-grade astrocytoma to high-grade glioblastoma, since p53 mutant cells are able to overtake normal p53 cells (Sidransky et al., 1992). The high frequency of numerous mutations in GBM, as well as in many others cancer types, suggest the existence of an intricate crosstalk link between the diverse nodes, which regulate cancer development and progression.

In addition, recent evidences suggest that several epigenetic mechanisms are important factors that are contributing to the pathogenesis of many different cancers, including GBM (Lee et al., 2017). Given the critical role of gene expression, several epigenetic modulators are being investigated as targets for developing new drugs against GBM, either to be used alone or in combination with other therapies (Lee et al., 2017; Chen et al., 2018), and also to be exploited as novel prognostic and predictive markers. In particular, histone deacetylase (HDAC) inhibitors are emerging as a promising class of anticancer drugs and are currently undergoing both preclinical and clinical trials as innovative GBM therapeutic agents (Suraweera et al., 2018). SGK1 (serum/glucocorticoid-regulated kinase 1) is another important survival kinase that regulates cell proliferation and differentiation (Kulkarni et al., 2018). Recent findings showed that SGK1 is involved in modulating autophagy and survival response to oxidative and reticulum stress, two factors contributing to the development of resistance to radiotherapy. Ortuso et al. identified a new SGK1 inhibitor, named SI113 that showed to be particularly efficient against GBM in cellular models both as a single agent and in combination with radiotherapy (Talarico et al., 2015, 2016).

Noteworthy, in recent years the discovery of novel immune strategies, especially checkpoint inhibitors, has paved the way on new appealing treatments for several cancer settings. The mode of action for checkpoint inhibitors is that they interfere with the immune escape mechanisms that are adopted by tumor cells to induce host immune system tolerance. Given that GBM is particularly associated with severe immunosuppression, it might be the ideal candidate for checkpoint inhibitor therapy (Huang et al., 2017). Indeed, several clinical trials of checkpoint inhibitors are ongoing in GBM, as well as in other brain carcinomas, either as single agents or in combination with conventional therapy (Buchbinder and Desai, 2016; Roth et al., 2016). Among these, Nivolumab is an anti-programmed cell death-1 (PD-1) monoclonal antibody that is currently undergoing the first large randomized clinical trial (NCT 02017717) (Filley et al., 2017). Even if these trials failed, the development of checkpoint inhibitors remains a promising alternative strategy against GBM that is currently being widely pursued.

Another important topic that is being widely investigated is the key role played by cancer stem cells (GSCs), which contribute to the high proliferative rate and to the infiltrative nature of GBM (Huang et al., 2017). When compared to the non-stem subpopulation, GSC subpopulation is innately resistant to chemoradiotherapy as a whole (Chen et al., 2012; De Bacco et al., 2016), and represents a prominent factor in GBM since GSCs appear to be involved in tumor generation, therapeutic resistance, and relapse (Bradshaw et al., 2016). As a matter of fact, GSCs possess the ability for long-lasting self-renewal and proliferation, thus giving birth to downstream progenitor cells with reduced differentiation, mitotic, and a high self-renewal potential, which, ultimately, lead to tumor growth (Bradshaw et al., 2016). As a consequence of such features, GSCs are becoming attractive targets to explore for the development of new chemotherapies.

Current pillars of GBM therapies include surgical resection and radiotherapy. Among chemotherapeutic agents, Temozolomide (TMZ), which was first introduced in the late 1990s, still remains as the drug of choice in GBM treatment. Recent research on TMZ has focused primarily on finding innovative and improved delivery systems when the drug is administered by itself (Khan et al., 2016, 2018; Lee, 2017) or in combination with other therapies (Lam et al., 2018). In particular, the combination with molecules that target HSP90 and HDAC was found to enhance the therapeutic effect of TMZ when used along with radiotherapy (Choi et al., 2014).

Today, several drugs are currently under investigation. Ongoing clinical trials include agents targeting RTK and signal transduction pathways, or antiangiogenic mechanisms through different methods, such as gene therapy, immunotherapy, reirradiation, radiolabeled drugs, and many others, alone or in combination. Unfortunately, to date the results from these trials have been quite disappointing. For instance, the first generation of EGFR inhibitors, gefitinib and erlotinib, had raised big expectations in GBM due to the success that they had achieved in lung cancer treatment. However, even though the alteration of EGFR is also found in brain cancer, these inhibitors failed in GBM clinical trials (Rich et al., 2004). Similarly, the experience with imatinib, an ATP binding site inhibitor of the PDGFR, KIT, and ABL kinases, was disappointing (Mellinghoff et al., 2011). Unsatisfactory results were also obtained with the mTOR inhibitor Rapamycin, which was considered one of the most advanced and promising agents in clinical development. However, when Rapamycin was subjected to clinical trials as single agent for PTEN deficient and recurrent GBM, the outcome was quite poor (Mendiburu-Elicabe et al., 2012). The overall disappointing results in studies performed with patients affected by high grade glioma, has been associated to an Akt feedback mechanism that leads to the reactivation of the entire PI3K pathway. Better results have come from the combination of rapalogs and EGFR kinase inhibitors, even though clinical trials for this therapeutic approach were also complicated by the need to reduce the doses of the drugs in order to attenuate toxicity (Kreisl et al., 2009; Reardon et al., 2010).

Accordingly, monotherapy strategies are often inadequate to achieve a powerful therapeutic intervention in GBM due to the lack of efficacy and the onset of severe side effects. In general the failure of the single agent approach may be caused by: (i) the activation of feedback compensatory mechanisms, which could lead to tumor cell resistance, and (ii) the deficiency of reliable predictive biomarkers that could possibly be helpful in selecting more sensitive patients for a given therapeutic approach (Khan et al., 2013). Consistently, radio and chemotherapy are widely used together in many solid cancers since they showed to be particularly potent when used in combination (Brunner, 2016); highlighting the fact that concurrent inhibition of different oncogenic proteins/networks could be a successful strategy for treating this form of cancer. In this context, nowadays, multi-targeted therapeutic strategies, by means of designing small molecules able to inhibit more than one specific molecular target, offer a promising alternative and have already been incorporated in new therapeutic drug design approaches for the development of anticancer drugs (Costantino and Barlocco, 2018; Ramsay et al., 2018). Indeed, in the last few years, a considerable number of compounds based on multitarget approach (MTA) have been described (Petrelli and Valabrega, 2009; Chen et al., 2013; Jiang et al., 2017).

In order to succeed in the development of multitarget agents, the design strategy must focus first on the ability to distinguish between healthy and cancer cells. To this aim, a ligand should be designed with the ability to preferentially hit a gene/protein network that is up-regulated in cancer cells, thus having a lower impact on the healthy ones. In this context, a multimodal agent capable of binding more than one specific target that is differentially expressed should be more efficient in affecting cancer cells, while having little effect on the cellular function of healthy cells (Xie and Bourne, 2015).

The increasing need for effective therapies in GBM, prompted us to focus this paper on examining another important approach in this field that entails the development of poli-functional preclinical strategies based on the use of small molecules, or combination of more than one agent, capable of targeting multiple nodes that are critical for GBM development and progression.

## The Multi-Targeted Strategy

**FIGURE 1:**
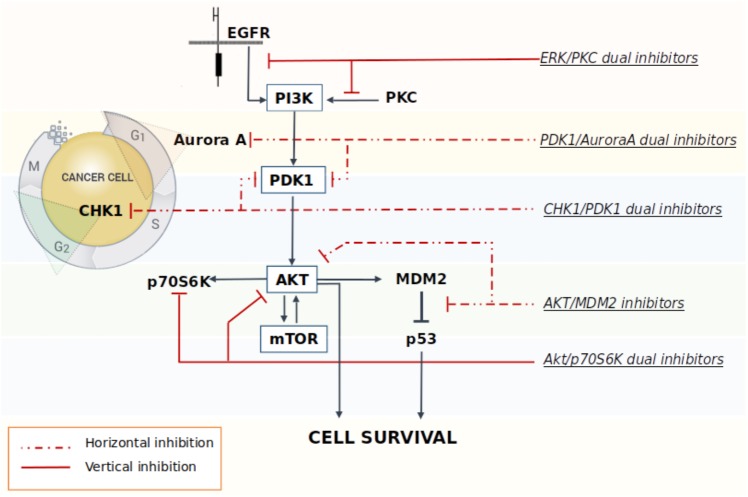
Schematic representation of some multitarget strategies investigated to promote the block of compensatory signaling mechanisms in cancer.

A multi-targeted strategy could be pursued through the co-administration of drugs targeting different key nodes of oncogenesis, or by merging different pharmacophores in a way to create new molecular entities that are capable of simultaneously and effectively hit different crucial nodes. However, one of the limitations of multimodal inhibition comes from the potential lack of selectivity against key targets of overexpressed networks that could turn the multitarget ligand into a promiscuous agent with a broad spectrum of activity, thus leading to the onset of off-target effects (Giordano and Petrelli, 2008). Although, the careful design of a ligand meant to specifically modulate several targets chosen *a priori* should in part alleviate this problem, in reality the dividing line between unwanted and desired effects is extremely thin (Handler et al., 2018). As a consequence, in order to overcome these drawbacks, the rational design of new multitarget agents for cancer treatment is now differentiated on the basis of the relationship between the crucial nodes that are being targeted by the drugs. In the *vertical inhibition* approach the molecular targets belong to the same cellular signaling axis, while in the *horizontal inhibition* approach the multitarget ligand interacts with different nodes of distinguished pathways (Yap et al., 2013) (**Figure 1**). Both strategies commonly attempt to block compensatory signaling mechanisms resulting in improved clinical benefits. For instance, Pitter et al. (2011) demonstrated that the vertical inhibition of Akt/mTOR pathway by the use of perifosine (Akt inhibitor) and temsirolimus (mTOR inhibitor) decreased tumor proliferation and induced apoptosis both *in vitro* and *in vivo* models of GBM, thus suggesting that the combination acts in synergy to inhibit the Akt/mTOR axis. In regards of the horizontal strategy, the dual targeted inhibition of MEK and PI3K pathway effectors promises to be a valid strategy to overcome resistance to MEK inhibitor therapy in metastatic colorectal cancer, which is characterized by a frequent perturbation of the MAPK and PI3K signaling axis (Temraz et al., 2015). Indeed, recent studies have shown that the dual targeted inhibition of MEK and PI3K pathway effectors has an enhanced efficacy against mutated colorectal cancer with respect to treatment using a single agent.

### Co-administration

#### Akt/mTOR and MDM2/mTOR Pathways

Many studies have shown that the Akt/mTOR pathway plays a key role in the pathogenesis of cancer, including GBM (Gulati et al., 2009; Jhanwar-Uniyal et al., 2015). GBM typically expresses p53 with a wild-type amino acid sequence; the reactivation of p53 functionality in cancer cells can be achieved through the blockade of the oncogenic inhibition caused by the AKT/mTOR pathway, which in turn, triggers the undesired excessive stimulation of MDM2. In this context, the simultaneous targeting of both the AKT and p53 axes proved to be particularly effective in cancer cells. A study performed in acute lymphoblastic leukemia cell lines showed that the inhibition of the AKT pathway synergizes with the MDM2 inhibitor Nutlin-3 to induce p53 reactivation and, consequently, cancer cells apoptosis (Zhu et al., 2008). Analogously, the concomitant administration of the mTOR inhibitor Everolimus and Nutlin-3 induces a synergistic inhibition of GBM cells and GSCs viability (Daniele et al., 2015). Preliminary studies also confirmed the presence of a synergic antiproliferative activity in GBM cell lines after cotreatment with the novel mTOR/AKT inhibitor FC85 and the MDM2/p53 blocker ISA27 (Daniele et al., 2015). The experimental data showed that this combination reactivated the p53 pathway, which was accompanied by a synergistic inhibition on U87MG cell viability. The same effect has also been observed in U87MG derived stem cells, thus resulting in improved apoptosis and a significant promotion of stem cells differentiation. The antitumor synergy elicited by the vertical inhibition of these two targets was also observed in a preclinical animal model of liposarcomas (Laroche et al., 2017). Results show a significant increase in apoptosis induced by the combination of the two drugs with respect to treatment using a single agent. Moreover, the co-administration of the MDM2 antagonist RG7388 and the dual inhibitor PI3K/mTOR BEZ235 was also able to significantly reduce tumor growth rate, thus indicating that the combination strategy designed to inhibit the AKT/mTOR signaling and to re-activate p53 signaling at once, may be potentially effective in different cancer types, including GBM.

#### PI3K/CDKs

In a paper published by Cheng et al. (2012), investigated the possibility to simultaneously inhibit the lipid kinase PI3K and the Cyclin-dependent kinases CDK1 and CDK2. PI3K is known to block proliferation rather than induce apoptosis, and this is probably one of the main reasons for the failure of using PI3K inhibitors as monotherapy. Cheng et al decided to study the effects induced by the combination of PI3K inhibition with compounds able to target CDK1/2, which are also important hallmarks in many cancers. PIK-75 (Hayakawa et al., 2007a,b), initially discovered as a PI3Kα inhibitor, is also able to potently induce apoptosis in glioma. The study performed by Cheng showed that this compound has a multitarget (PI3K and CDK1/2) profile. The blockade of CDK2 cooperates with the inhibition of PI3K to drive apoptosis. Roscovitine is another kinase inhibitor currently in trials for the treatment of solid tumors, with a potent activity against CDK1 and CDK2 (Benson et al., 2007; Hsieh et al., 2009). Results demonstrate that CDK1/2 inhibitors, siRNAs and roscovitine, in association with the PI3K inhibitor PIK-90 drive the cells to programmed death trough the blockade of the antiapoptotic protein Survivin. Roscovitine given in combination with PIK-90 is well tolerated *in vivo* and was found to induce apoptosis also in human glioblastoma xenografts. In conclusion, the inhibition of PI3K, CDK1, and CDK2 together can convert a cytostatic therapy due to PI3K inhibition into an apoptotic one. These results offer a preclinical rationale to evaluate this therapeutic strategy in glioma patients.

### Multitarget Ligands (MTDL)

#### PDK1/Aurora A

The Akt/PDK1 and AurA signaling pathways play a pivotal role in GBM cellular survival/migration and in the self-renewal of the GSCs. The dual inhibition of these targets represents an innovative medicinal chemistry approach and few molecules in literature showed the ability to hit both targets at once. Our laboratory recently investigated the effect of the co-administration of two selective inhibitors of PDK1 (MP7) and AurA (Alisertib), compared to the treatment with a single new multitarget inhibitor, namely SA16 (Sestito et al., 2016), which proved to inhibit simultaneously both PDK1 and AurA kinases, with IC_50_ values in the low nanomolar (416 and 35 nM, respectively) (Daniele et al., 2017). Computational studies were also performed to gain insights into the binding mode of SA16 against the PDK1 and AurA kinases. The results from these studies suggest that SA16 binds to the DFG-out (i.e., allosteric) conformation of PDK1. This novel dual inhibitor showed the ability to block cell proliferation, reduce tumor invasiveness, and trigger cellular apoptosis in U87MG, ANGM-CSS, and U343MG cell lines. Moreover, the new AurA/PDK1 dual-target molecule SA16 showed significant efficacy against U87MG-derived stem cells, inducing their differentiation and apoptosis. Taken together these results show that the dual PDK1/AurA inhibition offers an innovative and very promising multitarget strategy for GBM therapy. In addition, the ability of this double kinase inhibitor to also deplete GSC subpopulation, will further improve its efficacy in the treatment of this severe disease.

#### PDK1/CHK1

In a study by Signore et al. (2014), the authors investigate the effect induced by the staurosporine derivative UCN-01 in diverse collection of GSC lines. Initially published as specific inhibitor of PKC, UCN-01 was later discovered to have the ability to inhibit multiple kinases: it is a potent inhibitor of CHK1 (Ki = 5.6 nM), PDK1 (IC50 = 5.0 nM), PKCβ (IC50 = 10 nM), and CDKs (Ki: CDK1 and CDC2 95 nM, CDK2 30 nM, and CDK4 3.6 mM), and of other PKC isoforms that are inhibited with a lower potency. UCN-01 activity was demonstrated both *in vitro* on glioma cell lines and *in vivo* on U87MG xenografts (Davies et al., 2000; Zhao et al., 2002; Komander et al., 2003; Gani and Engh, 2010). Signore showed how the simultaneous multipathway inhibition by UCN-01 significantly slows down the growth of GSCs *in vitro*. These results were also confirmed in both orthotopic and heterotopic GBM *in vivo* models. Additional investigations on the molecular and functional effects of UCN-01 revealed that the sensitivity to this agent is associated with the activation of PDK1 and CHK1. The authors then suggest that a combined inhibition of PDK1, which mediates survival signals, and CHK1, which initiates DNA damage response, could be a potentially effective therapeutic approach to target GCSs, thus reducing growth of human GBM (Signore et al., 2014).

#### Akt/p70S6K

M2698 is an orally bioactive, potent, selective dual inhibitor of p70S6K and Akt, currently in phase I clinical trials for patients with advanced malignancies (ClinicalTrials.gov identifier: NCT01971515) (Machl et al., 2016). M2698 demonstrated a high potency both *in vitro* (IC50 = 1 nM for p70S6K, Akt1, and Akt3 inhibition; IC50 = 17 nM for pGSK3β indirect inhibition) and *in vivo* (IC_50_ = 15 nM for pS6 indirect inhibition). M2698 also revealed a fairly selective activity, since only six out of 264 kinases had an IC_50_ within 10-fold of p70S6K. Both *in vitro* and *in vivo* investigations indicate that M2698 effectively induces a dose-dependent inhibition of p70S6K substrate phosphorylation, which provides a potent PI3K/Akt/mTOR pathway blockade. Moreover, it simultaneously targets Akt, thus overcoming the compensatory feedback loop. M2698 demonstrated the ability to cross the BBB, reduce tumor growth and extend survival in an orthotopically implanted model of (human) U251 GBM. Recent studies prove that this compound is also able to inhibit tumor growth in mouse breast xenograft models derived from PI3K/Akt/mTOR pathway-dysregulated cell lines (MDA-MB-453 and JIMT-1) (Machl et al., 2016).

#### EGFR/PKC

**FIGURE 2:**
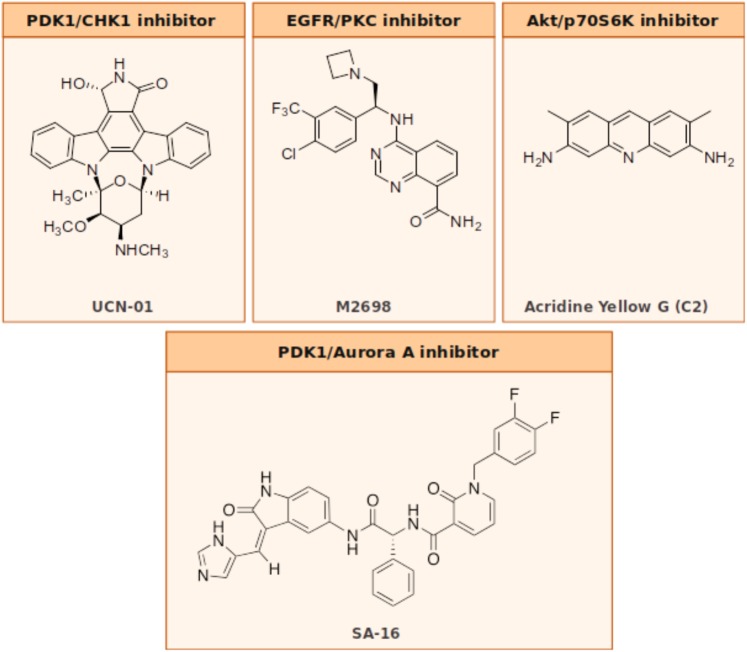
Dual inhibitors’ chemical structures.

Acridine yellow G is a yellow staining agent emitting a strong bluish-violet fluorescence that belongs to the acridine family of chemical compounds characterized by a nitrogen tricyclic scaffold. Acridine-based compounds have shown a wide variety of therapeutic properties, including antibacterial, antimalarial, and antitumoral. Among the acridine tricycle heteroatomic compounds, acridine yellow G (3,6-diamino-2 7-dimethylacridine, **Figure 2**) showed to be the most promising of the series for anti-GBM therapy. When tested in U87MG cell lines, acridine yellow G directly inhibits the kinases EGFR and PKCs with IC50 values of ∼7.5 and 5 μM, respectively, consequentially blocking the mTOR signaling and triggering the cell cycle arrest in the G_1_ phase, and, in turn, activating the apoptotic process in tumors. In particular, acridine yellow G preferentially blocks cell proliferation of the most malignant U87MG/EGFRvIII cells (PTEN-deficient U87MG glioblastoma cells that overexpress EGFRvIII) over the less malignant PTEN stably transfected U87MG cells (Qi et al., 2012). *In vivo* studies indicated that Acridine yellow G has the ability to induce a reduction of the tumor volumes in both subcutaneous and intracranial mice models. Toxic effects in animals subjected to chronic treatment were undetectable. Globally, results indicate that Acridine yellow G is a safe and effective therapeutic agent for the treatment of aggressive gliomas, as well as other types of human cancers, such as lung cancer, that are also inhibited by this compound (Qi et al., 2012).

## Conclusion

Over the years, the pathophysiological properties of GBM have been extensively studied in order to find new potential targets useful for developing innovative and successful therapies. However, despite the progress made in our understanding of the disease and the development of new therapies, the patient’s prognosis still remains poor. Many therapeutic strategies adopted in the last decades, led to the development of various new agents capable of hitting nodes that are crucial for the survival of GBM. Among new treatments, those based on monotherapy have generally failed to meet the initial expectations, raising the notion that a strategy capable of targeting multiple kinases should be more effective in attacking this disease.

It is also important to note that intrinsic or acquired resistance continues to be one of the main obstacles to overcome in both single and multitarget approaches. Indeed, many factors are involved in the mechanism of resistance in GBM, including: (i) an increase in the migration of cancer cells, angiogenesis, and proliferation, (ii) a reduction in the sensitivity to apoptosis resulting from the expression of antiapoptotic regulatory proteins, and (iii) an increase in both drug efflux expression and molecular proliferation pathways, such as Akt and NF-kB signaling (Garner et al., 2013). In particular, for a single agent treatment, drug-resistance is mainly caused by the activation of compensatory mechanisms, or the acquisition of genomic and/or epigenomic changes. On this basis, it seems logical that a multitarget approach using the right mechanism-based combination of different targeted therapies might be the able to delay, or even overcome, drug resistance. Further pre-clinical and clinical investigations are needed to verify whether these multimodal approaches represent a valid strategy to ultimately circumvent drug resistance (Groenendijk and Bernards, 2014).

In conclusion, the simultaneous inhibition of different cellular pathways involved in cancer development is emerging as the new promising strategy to achieve clinically meaningful tumor regression and to limit the ability of cancer cells to develop escape mechanisms, which would lead to the onset of chemoresistance. Among these strategies being pursued are those that involve: (i) a combination of multiple selective inhibitors directed on the same pathway, but different targets, (ii) the simultaneous blockade of different key proteins of the cross-talked signaling pathways, or (iii) a multidirectional inhibition on different oncoproteins throughout distinct pharmacological approaches (e.g., combining lipid and cyclin-dependent kinases). All of these strategies could represent a valid approach in GBM therapy, especially when targeted to the genetic pattern of the patient.

Finally, we also need to highlight the very promising concept of synthesizing single molecular entities suitably designed to hit multiple targets at once and with an adequate ADMET profile. When compared to the co-administration of multiple drugs, single multitarget molecules could present several advantages, including a lower risk of drug–drug interaction, an improved bioavailability, a reduced susceptibility to adaptive resistance and a better pharmacokinetic profile.

Our goal in writing this paper was to explore the main trends in preclinical investigation, with a special focus on those therapeutic approaches that simultaneously target multiple oncoproteins at once, as these have shown the best potential of being successful against GBM.

## Author Contributions

SS and MR were responsible for review of the literature. SS, GC, MT, and SR wrote the manuscript. MR drawn figures. SR designed the study and contributed with valuable discussion and revision of the manuscript.

## Conflict of Interest Statement

SR and SS are inventors of two patents related to multitarget kinases inhibitors. The remaining authors declare that the research was conducted in the absence of any commercial or financial relationships that could be construed as a potential conflict of interest.
